# Pushing the Limits of Lateral Flow Immunoassay by Digital SERS for the Ultralow Detection of SARS‐CoV‐2 Virus

**DOI:** 10.1002/smsc.202400259

**Published:** 2024-08-10

**Authors:** Lara González‐Cabaleiro, Carlos Fernández‐Lodeiro, Lorena Vázquez‐Iglesias, Pablo Soriano‐Maldonado, Mark J. van Raaij, Gustavo Bodelón, Jorge Pérez‐Juste, Isabel Pastoriza‐Santos

**Affiliations:** ^1^ CINBIO Universidade de Vigo, Campus Universitario As Lagoas Marcosende Vigo 36310 Spain; ^2^ Departamento de Química Física Universidade de Vigo, Campus Universitario As Lagoas Marcosende Vigo 36310 Spain; ^3^ Centro Nacional de Biotecnología (CNB‐CSIC) calle Darwin 3 Madrid 28049 Spain; ^4^ Faculty of Experimental Sciences Universidad Francisco de Vitoria (UFV) Pozuelo de Alarcón Madrid 28223 Spain; ^5^ Departamento de Biología Funcional y Ciencias de la Salud Universidade de Vigo, Campus Universitario As Lagoas Marcosende Vigo 36310 Spain

**Keywords:** digital SERS, NanoBiosensor, SARS‐CoV‐2, SERS‐based LFIA

## Abstract

Lateral flow immunoassays (LFIAs) are easy‐to‐use antigen tests that provide different signal readouts, with colorimetric readouts being the most commonly used. However, these analytical devices have relatively low sensitivity and produce semiquantitative results, limiting their diagnostic applications. Herein, we address these challenges by implementing a digital surface‐enhanced Raman spectroscopy (SERS)‐based LFIA for the accurate and ultrasensitive quantitative detection of SARS‐CoV‐2 nucleocapsid (N) protein. Compared with average SERS intensity measurements, the digital approach allowed to overcome fluctuations in Raman scattering signals, thereby increasing the analytical sensitivity of the assay. Our method exhibited a quantification range of the viral protein in nasal swabs from 0.001 to 10 pg mL^−1^, and a limit of detection down to 1.9 aM (0.9 fg mL^−1^), improving colorimetric LFIAs and conventional‐SERS‐based LFIAs by several orders of magnitude. Importantly, this approach shows an analytical sensitivity of 0.03 TCID_50_ mL^−1^, which is greater than that reported by other immunoassays. In conclusion, we successfully demonstrate the robust detection and quantification of SARS‐CoV‐2N protein in nasal swabs at ultralow concentrations. The improvement in the sensitivity of LFIA by digital SERS may pave the way to translate this technology into the diagnostic arena for the ultrasensitive detection of microbes and disease biomarkers.

## Graphical abstract

A digital surface‐enhanced Raman spectroscopy (SERS)‐based LFIA for the accurate and ultrasensitive quantitative detection of SARS‐CoV‐2 nucleocapsid (N) protein is implemented. The method shows limits of detection down to 1.9 aM (0.9 fg mL^−1^), improving conventional‐SERS‐based LFIAs by several orders of magnitude. Importantly, this approach shows an analytical sensitivity of 0.03 TCID_50_ mL^−1^, which is greater than that reported by other immunoassays.

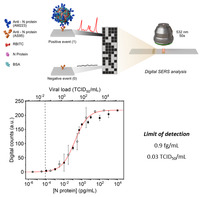

## 1 Introduction

The lateral flow immunoassay (LFIA) is a fast, reliable, and easy‐to‐use technology designed for on‐site testing.^[^ ^1^ ^]^ Conventional LFIAs typically employ antibody‐functionalized gold nanoparticles (AuNPs) as optical labels for antigen detection and colorimetric readout. In this assay, a fluid sample bearing the target analyte and AuNPs migrates by capillary force through a membrane that contains antibodies or antigens immobilized in test and control lines.^[^ ^2^ ^]^ A positive reaction leads to the concentration of the AuNPs in the detection area yielding a visual color change that can be assessed by the naked eye owing to the localized surface plasmon resonance of the nanoparticles.^[^ ^3^ ^]^ Owing to their operational simplicity and low cost, the use of LFIAs has rapidly extended from diagnostic applications to other fields such as environmental monitoring, drug testing, and food safety control among many others, with an estimated global market in 2019 of about US $5.98 billion.^[^ ^1^ ^]^

However, limited by the weak optical signal usually rendered by AuNPs, colorimetric LFIAs typically exhibit a relatively low analytical sensitivity as compared to the gold standard immunoassay enzyme‐linked immunosorbent assay (ELISA) and polymerase chain reaction (PCR).^[^ ^4^ ^]^ Therefore, when target analytes are present at low concentrations, such as in clinical samples, colorimetric LFIAs may not provide sufficient sensitivity, thereby restricting their applications. Hence, the growing demand for highly sensitive and quantitative methods for on‐site detection calls for new strategies to increase their sensitivity, particularly when trace analysis is sought.^[^ ^5^ ^]^ A prominent avenue focuses on increasing the signal intensity by improving the optical properties of the plasmonic labels,^[^ ^6^ ^]^ by chemical enhancement strategies,^[^ ^4^ ^]^ by employing fluorescent,^[^ ^7^, ^8^ ^]^ magnetic,^[^ ^9^, ^10^ ^]^ and photothermal labels,^[^ ^11^ ^]^ as well as by surface‐enhanced Raman scattering (SERS)^[^ ^12^, ^13^, ^14^ ^]^ among others.^[^ ^4^ ^]^ The analytical performance of different approaches developed for this purpose has been reviewed elsewhere,^[^ ^4^, ^14^ ^]^ being SERS one of the most sensitive, achieving limits of detection (LODs) ranging from 10^−9^ to 10^−15^ M.^[^ ^4^ ^]^

SERS is a vibrational spectroscopy that utilizes plasmonic metal nanoparticles as optical enhancers and is characterized by its extremely high sensitivity, unmatched multiplexing capabilities, and excellent photostability.^[^ ^15^, ^16^ ^]^ LFIAs based on SERS employ a class of optical labels known as SERS‐tags, which typically consist of plasmonic NPs encoded with intrinsically strong Raman scattering molecules and conjugated with antibodies.^[^ ^17^, ^18^ ^]^ Normally, the SERS‐based LFIAs are performed by measuring the average intensity of Raman scattering signals recorded on the test line via individual points or area scans (i.e., SERS mappings).^[^ ^19^, ^20^, ^21^ ^]^ Compared to colorimetric labels, SERS tags can substantially increase the signal sensitivity by several orders of magnitude.^[^ ^13^ ^]^ Hence, recent years have witnessed the rise of a plethora of SERS‐based LFIAs for the detection of an ample array of biological targets such as prostate‐specific antigen,^[^ ^22^ ^]^ pneumolysin,^[^ ^21^ ^]^ Alzheimer's disease‐related proteins,^[^ ^23^, ^24^ ^]^ pathogenic bacteria,^[^ ^25^ ^]^ or respiratory viruses^[^ ^26^, ^27^ ^]^ among many others.^[^ ^28^ ^]^ Furthermore, the highly resolved spectral bands of Raman emission (≈2 nm), much narrower than fluorescence emission bands (30–50 nm), facilitate the simultaneous detection of multiple targets.^[^ ^29^, ^30^ ^]^ This feature was leveraged for the simultaneous (i.e., multiplex) SERS detection and quantification of different analytes in a single test line including veterinary drugs,^[^ ^31^ ^]^ pathogenic bacteria,^[^ ^32^ ^]^ mycotoxins,^[^ ^33^ ^]^ and food allergens.^[^ ^34^ ^]^ Moreover, unlike the issue of photobleaching often encountered in luminescence and fluorescence assays, SERS provides the advantage of inherent high photostability of the Raman probes.

Reliable ultrasensitive detection and quantification are critical for early detection and diagnosis of many diseases, patient outcomes, and therapy response.^[^ ^35^ ^]^ Indeed, the concentrations of biomarkers in biological fluids at early stages of disease fall below pM down to aM level, and the physiological dynamic range of the human plasma proteome may vary within up to 10 orders of magnitude.^[^ ^36^ ^]^ In this context, the detection of proteins at very low concentrations for early diagnosis employing conventional methods is challenging, and thus new approaches are needed.

Digital bioassays featuring single molecule detection sensitivity have emerged as key technologies foreseen to revolutionize precision medicine and clinical practice.^[^ ^37^, ^38^ ^]^ Whereas average signals are quantified in conventional “analog” measurements, digital assays count individual signal events upon signal binarization into “1” or “0” (i.e., positive/negative) above a predetermined threshold value established to improve the filtering of the positive signals.^[^ ^39^ ^]^ Compared to analog measurements, digitalization reduces background noise and increases the sensitivity of the measurement at very low analyte concentrations,^[^ ^40^ ^]^ thereby offering a highly sensitive quantitative method for detecting rare targets in biological samples.^[^ ^41^, ^42^, ^43^, ^44^ ^]^ Recently, Brolo and collaborators developed a digital SERS approach for the ultrasensitive detection of enrofloxacin and ciprofloxacin at single molecule level.^[^ ^45^ ^]^ Later studies have confirmed that the digital SERS methodology yields higher sensitivity, accuracy, and robustness than conventional measurements of average Raman intensities.^[^ ^46^, ^47^, ^48^, ^49^, ^50^, ^51^, ^52^ ^]^

**Scheme 1:**
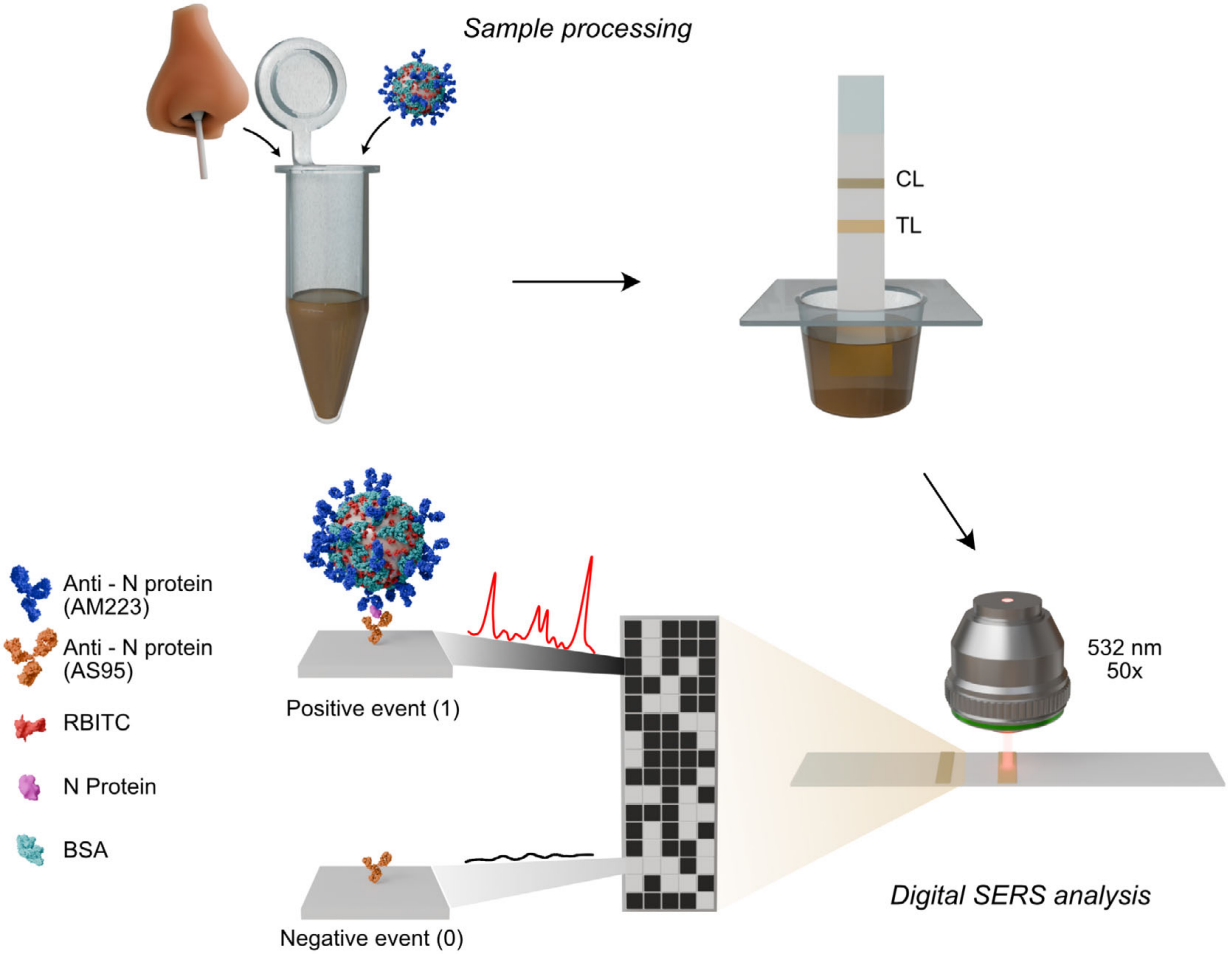
Schematic representation of the workflow of the digital SERS‐based LFIA proposed from sample collection to data recording. The nasal swab sample is first incubated with the SERS tags and then applied to the LFIA strip, which moves by capillary action toward the test zone containing the test (TL) and (CL) control lines bearing immobilized mouse monoclonal anti‐N protein antibodies and streptococcal protein G, respectively. In the presence of the viral antigen, the SERS‐tag‐N protein conjugate is immunocaptured in the test line and subjected to SERS employing a 532 nm laser line. Digital analysis is carried out by counting positive signal events recorded in a SERS mapping.

Herein, we report a digital SERS approach to increase the analytical sensitivity of current LFIAs for diagnostic applications. We focused this work on the detection and quantification of the nucleocapsid (N) protein of SARS‐CoV‐2. The N protein is commonly used as a biomarker of viral infection because it is the most abundant viral antigen and exhibits low mutation rates.^[^ ^53^ ^]^ Conventional (i.e., colorimetric) lateral flow tests provide reliable results only when large quantities of the virus are present in samples. Indeed, their sensitivity may drop to less than 50% for infected people with low viral titer leading to false‐negative results, which can increase the risk of virus transmission, questioning their utility to identify asymptomatic or early infected patients.^[^ ^54^ ^]^ Different groups have already explored the combination of SERS with LFIAs to improve SARS‐CoV‐2 detection.^[^ ^55^, ^56^, ^57^, ^58^ ^]^ In such studies, quantification is based on measuring the average intensity of a Raman‐active spectral feature in area scans (i.e., SERS mappings) performed in the test line of the lateral flow strip. Despite that the method is robust and reliable, the quantification capability of this approach may be challenged by high levels of background noise and non‐homogenous signal intensities at low antigen concentrations. To address this limitation, we implemented a digital analysis in a SERS‐based sandwich LFIA for ultrasensitive quantification of SARS‐CoV‐2 in nasal swabs. Our approach employs highly efficient SERS‐tags consisting of core‐shell Au@Ag nanoparticles codified with rhodamine B isothiocyanate (RBITC) and bioconjugated with monoclonal antibodies against the N protein (**Scheme** **1**). By this method, we have been able to detect SARS‐CoV‐2 N protein with an LOD of 0.9 fg mL^−1^ (1.9 aM), and viral particles down to 0.03 TCID_50_ mL^−1^, significantly improving colorimetric LFIAs and conventional‐SERS‐based LFIAs by several orders of magnitude.

## 2 Results and Discussion

### 2.1 Development of SERS‐Based LFIA. Digital SERS‐Based LFIA for the Detection of SARS‐CoV‐2N Protein

As optical nanoprobes, we synthesized RBITC‐encoded SERS tags because they exhibited an excellent performance in SERS‐based LFIAs previously developed by our group.^[^ ^21^, ^34^ ^]^ Spherical Au@Ag NPs were synthesized by a seed‐mediated growth employing iron(II) as a reducing agent, codified with RBITC, and conjugated with monoclonal antibodies against the N protein by physical adsorption (see Section 4). The as‐synthesized Au@Ag NPs (≈50 nm) feature relatively high monodispersity and a localized surface plasmon band centered at 430 nm. The codification of the particles resulted in high Raman intensity due to resonance enhancement effect upon illumination with a 532 nm laser line, yielding the characteristic Raman fingerprint of RBITC and exhibiting a strong intensity band at 1646 cm^−1^ ascribed to an aromatic C‐C stretching (Figure S1).^[^ ^21^, ^34^ ^]^

The principle of the SERS‐based LFIA developed herein relies on a non‐competitive sandwich approach with mouse monoclonal antibodies against N protein and streptococcal protein G bearing high affinity toward immunoglobulins^[^ ^59^ ^]^ printed on the test and control lines of the strip, respectively (Scheme 1). Non‐specific binding of SERS tags may lead to false positives and increased background noise, which will have a detrimental effect on the digital counting protocol. In this context, a key parameter that may be tuned to reduce non‐specific binding and thus optimize the analytical performance of LFIAs is the composition of the running buffer.^[^ ^4^ ^]^ To this aim, a solution consisting of running buffer and SERS tags was assessed by lateral flow dipstick in the absence of antigen (i.e., N protein). A running buffer composed of 3% w/v bovine serum albumin (BSA), 1% PVP w/v, and 0.5% v/v Triton X‐100 in PBS at pH 7.4 yielded the best results (Figure S2). In this assay, no significant Raman signals were detected in the SERS mapping recorded in the test line, indicating that the levels of non‐specific binding are negligible. The presence of the colored band in the control line demonstrates that the lateral flow assay was carried out correctly. It is important to note that no significant SERS signals were detected out of the test line, thereby confirming the absence of non‐specificity of this immunoassay.

**Figure 1:**
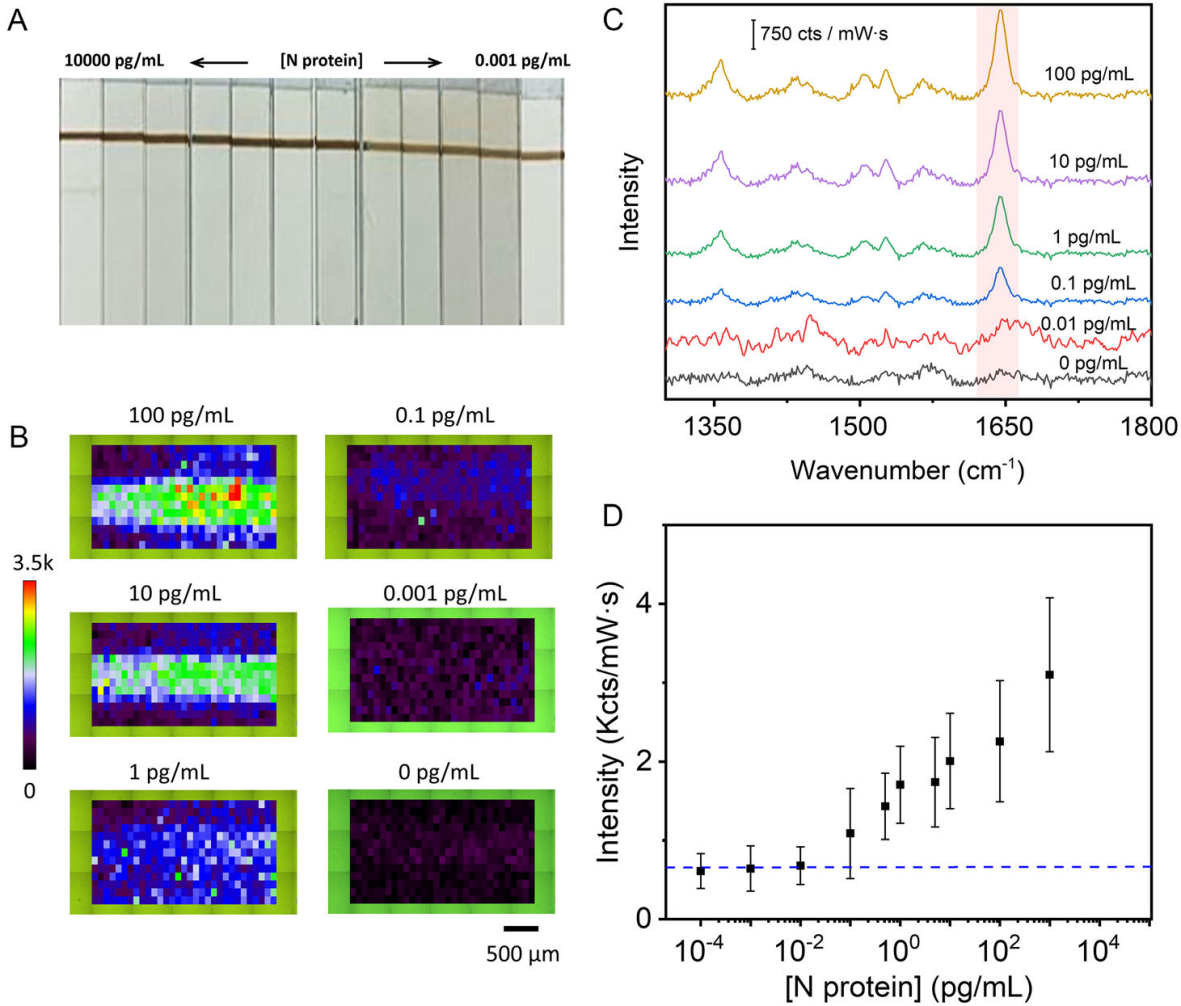
A) Photographs of different LFIA strips at varying N protein concentrations as indicated. B) Representative SERS mappings were measured at the test line of the different lateral flow strips using the peak intensity at 1645 cm^−1^. The scale bar represents 500 μm. All SERS measurements were carried out with a 532 nm laser line, 50× objective, 12.90 mW laser power, acquisition time 1 s, 3100 × 1300 μm^2^, 100 μm step size, and 403 points. C) Representative average SERS spectra measured in the test line in the presence of different N protein concentrations. The red‐shadowed region highlights the Raman peak at 1645 cm^−1^. D) Variation of the average SERS intensity at 1645 cm^−1^ with the N protein concentration. The error bars represent the standard deviation obtained from three independent measurements. The blue dashed line indicates 3 times the standard deviation of the background.

**Figure 2:**
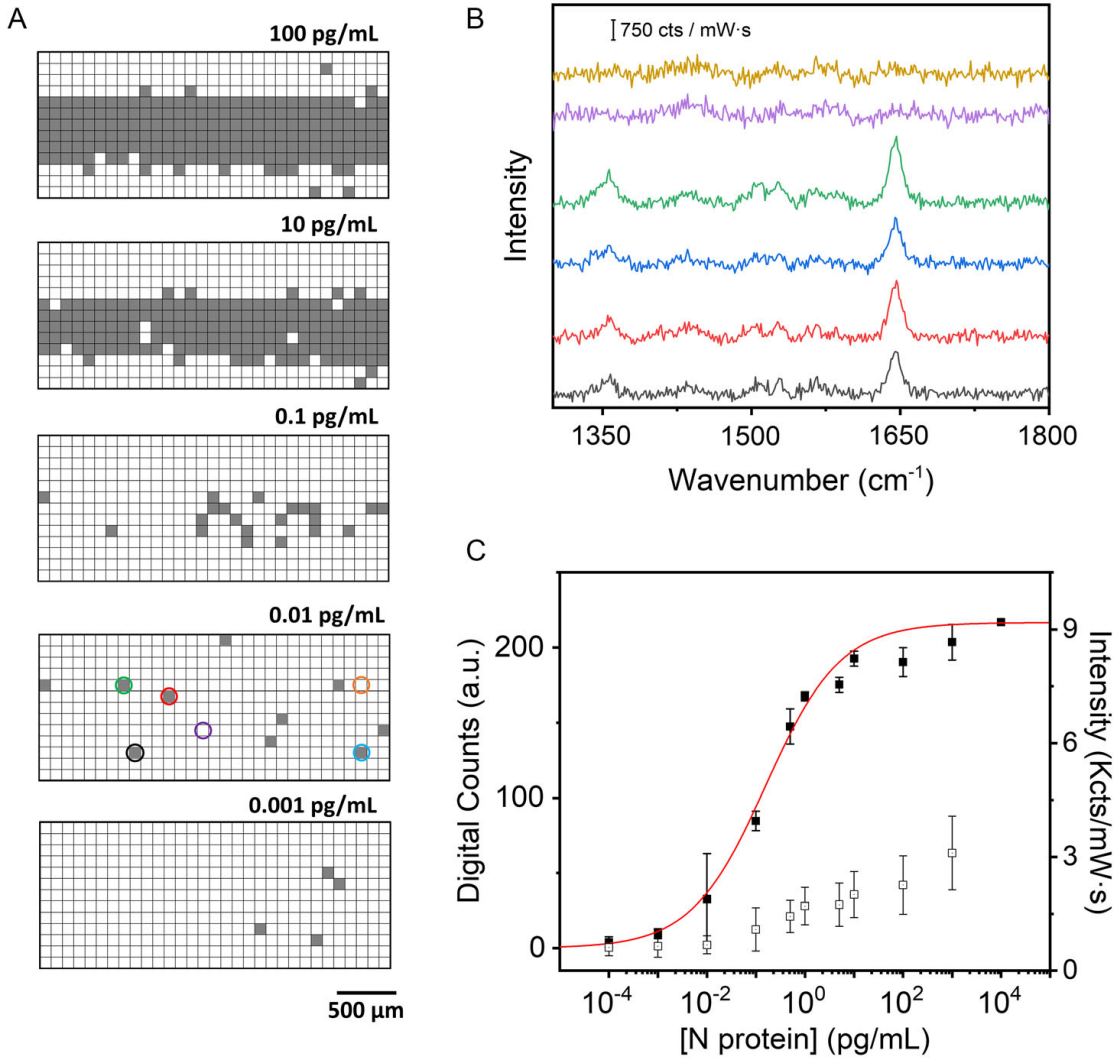
A) Digital maps were obtained for the different N protein concentrations as indicated. The gray and white squares correspond to positive and negative events, respectively. B) Representative SERS spectra of positive and negative events showing the presence or the absence of the characteristic Raman peak at 1645 cm^−1^, the color code of the spectra corresponds with the pixels highlighted in the 0.01 pg mL^−1^ map. C) Variation of the digital SERS counts with the concentration of N protein (black squares). The red curve represents the best fitting of a four‐parameter logistic (4PL) equation to the experimental data. As a guide, the measured average SERS intensity at 1645 cm^−1^ with the N protein concentration is also plotted (open squares). Each point represents the mean of the three replicates per concentration with its corresponding standard deviation. SERS measurements were obtained with a 532 nm laser line, 50× objective, 12.90 mW laser power, 1.0 s acquisition time, 3100 × 1300 μm^2^, 100 μm step size, and 403 points.

We first sought to develop the digital assay focusing on the detection of recombinant N protein spiked in nasal swabs obtained from healthy volunteers (see Section 4). The solution containing N protein at concentrations ranging from 0.1 μg mL^−1^ to 0.1 fg mL^−1^ as well as a blank sample with no protein was incubated with the SERS tags, mixed with running buffer, and subsequently run on the test strips (in triplicates for each concentration, see Section 4). Only the highest concentration of protein (0.1 μg mL^−1^) is detected by visual inspection of the test line, while the control line presents a uniform color among all concentrations assessed, as expected (**Figure** **1**A). Next, SERS mappings were acquired on the test line of the different lateral flow dipsticks over a 3100 × 1300 μm^2^ area (403 points, 100 μm step size), employing the peak intensity at 1645 cm^−1^. The recorded SERS mappings show that the SERS signal in the scanned area decreases with decreasing N protein concentrations (Figure 1B). The “analog” calibration curve generated relating average SERS intensities to each antigen concentration yielded an LOD of 0.1 pg mL^−1^ (**Figure** **2**C,D). This value is 4‐5 orders of magnitude greater than that obtained by the colorimetric approach (10^3^–10^4^ pg mL^−1^ as determined by three different commercially available colorimetric tests, see Figure S3), thereby showcasing the substantial improvement in sensitivity achieved through conventional SERS.

Next, to implement the digital analysis, the recorded SERS mappings (Figure 1B) were transformed to the binary format by setting a specific threshold based on the average signal‐to‐baseline area between 1620 and 1660 cm^−1^ measured at the control line without antigen, plus 3 times the standard deviation above the mean measured in the control test without antigen.^[^ ^45^, ^51^ ^]^ The pixels with signal‐to‐baseline areas between 1620 and 1660 cm^−1^ above the threshold were assigned to 1, whereas those equal or below were set to 0. More detailed information on the data treatment and its analysis is presented in Figure S4.

Figure 2A shows the digitized version of the SERS mappings generated for various N protein concentrations, as indicated, representing the positive “1” and negative “0” events in gray and white, respectively. As expected, the number of positive events gradually decreases with the analyte concentration. Noteworthy, the digital SERS map recorded in the blank sample is completely white (data not shown), thereby indicating the absence of any signal. Figure 2B shows representative positive and negative SERS spectra measured in the test line at 0.01 pg mL^−1^ of N protein. It should be noted that the digital SERS maps from the control line consistently show a roughly constant value of positive events (220 ± 5).

A digital calibration curve was generated by plotting the number of digital counts versus the different N protein concentrations (Figure 2C). As expected, the variation of the digital counts follows a sigmoidal‐shaped curve. This behavior can be described by the 4PL equation (mathematically similar to the Hill equation), commonly employed in immunoassays,^[^ ^60^ ^]^ and represented by

(1) $$y=A_{1}+\frac{\left(A_{2}-A_{1}\right)X^{p}}{X_{0}^{p}+X^{p}}$$

where *y* is the number of digital counts and *X* is the *N* protein concentration. *A*_1_ and *A*_2_ are the values of the lower and upper asymptote, respectively, *p* is the slope at the inflection point (also known as the Hill coefficient), and *X*_0_ corresponds to the value of *X* corresponding to 50% of the maximum asymptote.^[^ ^60^ ^]^ The best fit of the 4PL equation to the experimental data yielded the red curve in Figure 2C and the following values for the parameters: *A*_1_ is the value of digital counts of the control as deduced from the negative samples and equal to 0, *A*_2_ = 217 ± 2 (in agreement with that measured in the control line), *X*_0_ = (1.6 ± 0.3) × 10^−1^ pg mL^−1^, and *p* = 0.57 ± 0.06.

A value of *p* smaller than one indicates negatively cooperative binding, which is an expected behavior since the probability of binding reduces as the surface coverage increases. To estimate the LOD from a 4PL model, it is commonly determined by adding three times the standard deviation of the background noise to the mean signal of the blank samples. In our case, since the digital SERS value for the blank samples was consistently 0, we have chosen a digital SERS value of 3 as the criteria to estimate the LOD. Interpolating this value using the 4PL equation resulted in an LOD for N protein spiked in nasal swabs of 0.9 fg mL^−1^, which is two orders of magnitude lower than the previously described conventional analog SERS approach (0.01 pg mL^−1^, Figure 1). Additionally, the method provides a dynamic range that allows quantification of the N protein over a concentration range of five orders of magnitude (from 0.001 to 10 pg mL^−1^).

Other groups have also explored the use of SERS in LFIAs for the detection of N and spike (S) proteins of SARS‐CoV‐2 using average SERS intensity measurements. For instance, Lai and collaborators detected the N protein in spiked saliva at an LOD of 0.03 pg mL^−1^ employing Au/Ag core‐shell nanoparticles encoded with mercaptobenzoic acid (MBA).^[^ ^61^ ^]^ In another work, two‐dimensional Ag/black phosphorus (Ag/BP)‐Rhodamine B nanosheets were used to achieve an LOD of 0.5 pg mL^−1^ in lysis buffer spiked with the N protein.^[^ ^62^ ^]^ Serebrennikova and collaborators detected the viral S protein diluted in buffer with an LOD of 0.1 ng mL^−1^ employing MBA‐modified spherical gold nanoparticles.^[^ ^55^ ^]^ Alternatively, Wang and collaborators reported a digital SERS‐based LFIA to detect the N protein in buffer at an LOD of 12 pg mL^−1^ using Au/Ag core‐shell nanoparticles codified with MBA as SERS tags.^[^ ^51^ ^]^ The digital counting approach has been also implemented in other SERS‐based immunoassay formats (not lateral flow) for the detection of the S protein of the virus. In this regard, the Brolo group reported an LOD of 6.3 ng mL^−1^ using core‐shell Au^4−MBA^@Ag nanoparticles to detect the antigen in diluted saliva.^[^ ^46^ ^]^ Additionally, through a digital SERS‐based approach, Shim and collaborators detected the viral S protein in buffer with an LOD down to 19 fg mL^−1^ employing bumpy core‐shell Au nanoprobes encoded with 4‐nitrobenzenethiol.^[^ ^50^ ^]^ Thus, in terms of analytical sensitivity, our approach significantly outperformed the aforementioned SERS‐based biosensing platforms by several orders of magnitude in most of them (see Table S1 in Supporting Information).

### 2.2 Digital SERS‐Based LFIA for the Detection of SARS‐CoV‐2 Virus

Commonly, SARS‐CoV‐2 infected people undergo an incubation period followed by exponential replication leading to a steep rise in viral load, enhanced infectivity, and greater risk of transmission.^[^ ^63^, ^64^ ^]^ The gold standard for laboratory diagnosis and molecular detection of SARS‐CoV‐2 infection is quantitative reverse transcription PCR. This extremely sensitive technique can provide an indirect measure of viral load, but it does not allow the quantification (i.e., titer) of infectious (i.e., replication‐competent) viruses and therefore does not provide adequate information on the progression of the disease. Furthermore, positive results by PCR can last for several weeks after a patient has recovered from the disease.

The viral titer can be determined in vitro by quantifying the 50% tissue culture infectious dose (TCID_50_) mL^−1^.^[^ ^63^ ^]^ In this technique, susceptible mammalian cells are cultured in vitro and then exposed to dilutions of a patient sample, or a viral stock, to ascertain the quantity required to induce 50% cell death. In this regard, the World Health Organization (WHO) has recommended expressing the LODs of SARS‐CoV‐2 in vitro diagnostics (IVDs) in TCID_50_ mL^−1^ aiming to set the performance and efficacy requirements of IVDs (WHO/2019‐nCoV/Essential_IVDs/2021.1).^[^ ^65^ ^]^ It has been reported that the total number of infectious SARS‐CoV‐2 viral units per individual during peak infection during COVID‐19 can be from 10^5^ to 10^7^ TCID_50_ mL^−1^.^[^ ^66^ ^]^ Most commercial LFIAs for SARS‐CoV‐2 testing present an analytical sensitivity ranging between 10^2^ and 10^5^ TCID_50_ mL^−1^,^[^ ^67^, ^68^, ^69^ ^]^ which is insufficient to reliably detect early‐stage and presymptomatic patients with low viral concentrations.^[^ ^70^ ^]^ Concerningly, an important fraction of the infected population may be missed by assays with low analytical sensitivity, thereby undermining public health efforts and putting individuals at risk. Indeed, the WHO has defined that the ideal LOD of IVDs should be less than 10^2^ TCID_50_ mL^−1^ (WHO/2019‐nCoV/Essential_IVDs/2021.1).^[^ ^65^ ^]^

**Figure 3:**
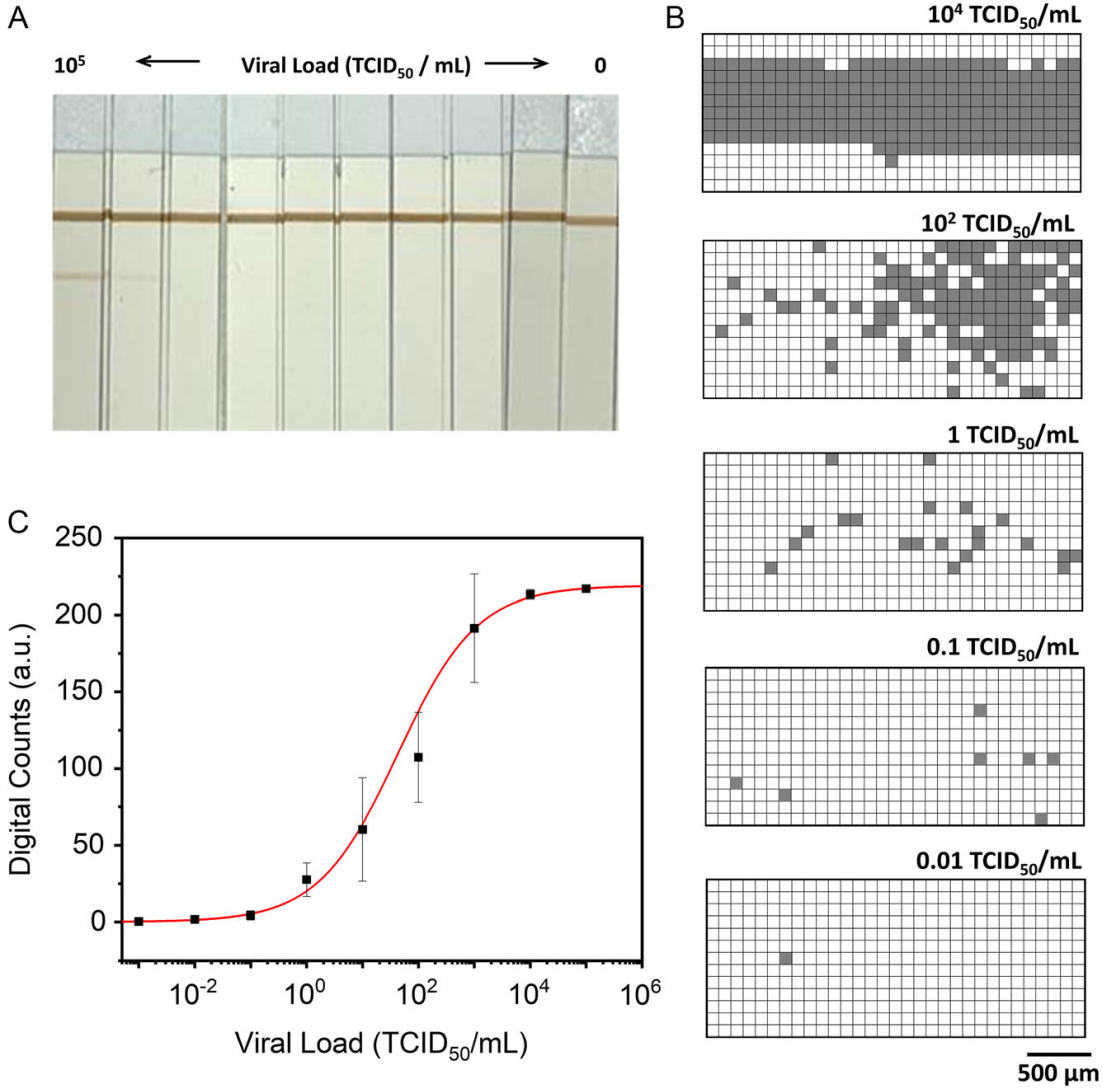
A) Photograph of LFIA strips probed with different viral loads (from 10^5^ to 0 TCID_50_ mL^−1^). B) Representative digital SERS maps after applying the threshold limit value to the signal to baseline SERS maps obtained at the indicated viral titers (TCID_50_ mL^−1^). C) Variation of the digital counts with the increasing of the viral load. Each black square represents the mean of three replicates with their corresponding standard deviation. The red line represents the 4PL calibration curve. All SERS measurements were obtained with a 532 nm laser line, 50× objective, 12.90 mW laser power, 1.0 s acquisition time, 3100 × 1300 μm^2^, 100 μm step size, and 403 points.

We next applied the digital SERS‐based LFIA method to assess whether we can further increase the LOD for the detection and quantification of SARS‐CoV‐2. To this aim, we employed inactivated virus (NR‐52287, BEI Resources) spiked in nasal swabs due to biosafety concerns. NR‐52287 (lot number 70033322) has an assigned value for the concentration of infectious virus of 2.8 × 10^5^ TCID_50_ mL^−1^ determined before inactivation. For the evaluation of the analytical sensitivity, serial dilutions of the viral stock from 1 × 10^5^ to 0.001 TCID_50_ mL^−1^, as well as a control assay performed with no virus, were subjected to SERS‐based lateral flow, performing triplicates for each concentration. As shown in **Figure** **3**A, the colorimetric signal in the test line is only detectable by the naked eye in the strips corresponding to 1 × 10^5^ and 1 × 10^4^ TCID_50_ mL^−1^, whereas the control line displayed similar color intensities in all samples tested, as expected (Figure 3A). The digital analysis evidenced that the number of positive events and the viral load are positively correlated, indicating a direct proportion between the amount of virus and the recorded digital SERS counts (Figure 3B). As for the N protein, a digital calibration curve was generated by plotting the digital counts versus TCID_50_ mL^−1^, showing a sigmoidal‐shaped trend (Figure 3C). The best fit of the 4PL equation to the experimental data yielded the red curve in Figure 3C and the following values for the parameters: A_1_ is the value of digital counts of the control as deduced from the negative samples and equal to 0, *A*_2_ = 219 ± 1, *X*_0_ = (43 ± 12) TCID_50_ mL^−1^, and *p* = 0.61 ± 0.03. From this regression curve, and considering a digital SERS value of 3 as the criteria to estimate the LOD, we obtained a value of 0.03 TCID_50_ mL^−1^, which is 3–4 orders of magnitude lower than that of commercially available colorimetric antigen test kits previously reported in the literature between 32 and 800 TCID_50_ mL^−1^,^[^ ^68^ ^]^ and 4 orders of magnitude than three commercially available colorimetric antigen test kits supplied from local drug stores and performed under the same conditions (see Figure S5 and methods section). Additionally, the method provides a dynamic range that allows quantification of the viral titer over four orders of magnitude (from 0.1 to 10^3^ TCID_50_ mL^−1^).

**Figure 4:**
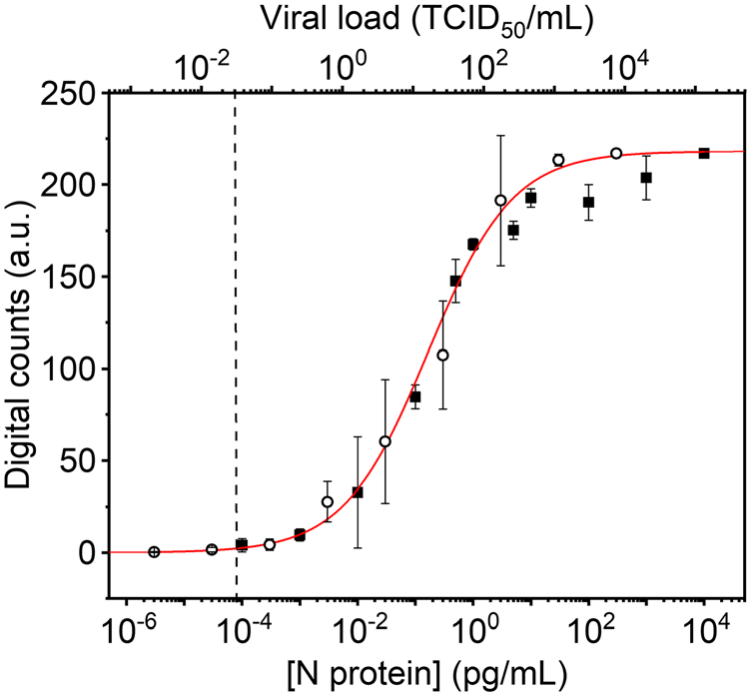
Correlation between the digital counts measured for the N protein concentration calibration (closed squares) and SARS‐CoV2 viral load (open circles). Each point represents the mean of three replicates per viral load or N protein concentration with its corresponding standard deviation. The dashed line indicates the estimated LOD; 0.9 pg mL^−1^ or 0.03 TCID_50_ mL^−1^.

Finally, we evaluated the relationship between viral titer (TCID_50_ mL^−1^) and N protein concentrations (pg mL^−1^) in spiked nasal swabs from the calibration curves shown in Figure 2C and 3C. Thus, if we consider that we can equate the estimated detection limits for protein N and the viral titer of 0.9 pg mL^−1^ and 0.03 TCID_50_ mL^−1^, respectively, it is possible to estimate the correspondence of TCID values in protein N concentration in  pg mL^−1^. **Figure** **4** shows a strong correlation between both parameters, thereby demonstrating the robustness and reliability of the digital SERS. Our results demonstrate that the digital SERS‐based LFIA achieves attomolar‐level sensitivity (0.9 fg mL^−1^ is equivalent to 1.97 aM assuming 47 kDa as the molecular weight of recombinant N protein), greatly surpassing the physiologically relevant concentration for SARS‐CoV‐2 detection (≈10 pM).^[^ ^50^ ^]^ We then compared the analytical sensitivity of the present sensing platform toward SARS‐CoV‐2 detection with the LOD reported for other antigen tests (Table S2 in the SI). As compared with the most sensitive immunoassays, the reported LOD for viral load (0.03 TCID_50_ mL^−1^) is one order of magnitude lower than S‐PLEX assay (0.36 TCID_50_ mL^−1^),^[^ ^71^ ^]^ SERS‐based microdroplet (0.32 TCID_50_ mL^−1^),^[^ ^72^ ^]^ and digital Simoa (0.29 TCID_50_ mL^−1^) (quanterix.com/wp‐content/uploads/2021/05/IFU‐0002v11_5.24.21). In regards to N protein, the LOD (0.9 fg mL^−1^) is 2 and 3 orders of magnitude higher than Simoa (99 fg mL^−1^) (quanterix.com/wp‐content/uploads/2020/12/SARS‐CoV‐2‐N‐Protein‐Advantage‐Data‐Sheet‐for‐HD‐X) and S‐PLEX (160 fg mL^−1^),^[^ ^71^ ^]^ respectively. Whereas the digital upconversion‐linked immunosorbent assay technology shows similar analytical sensitivity for viral load (0.08 TCID_50_ mL^−1^),^[^ ^73^ ^]^ its LOD toward N protein is substantially higher (330 fg mL^−1^). It should be noted that to experimentally validate this benchmarking, the reported method must be compared with others employing the same batch of inactivated viruses and antibodies.

Simoa and droplet digital ELISA are two digital bead‐based immunoassays where microwells and droplets, respectively, serve as partitions to physically contain single fluorescent beads as signals. These signal‐based compartmentalization approaches are known for their high multiplexing capabilities. However, the limited encoding capacity of fluorescent beads, signal interference, and a low bead analysis rate currently hamper their multiplicity. Noteworthy, the bead loading efficiency and entrapment rate significantly affect the detection accuracy of both methods due to high Poisson noise.^[^ ^74^ ^]^ Moreover, these complex methods require long imaging times of up to 30 min,^[^ ^75^ ^]^ whereas the acquisition time of a SERS mapping in the LFIA test line takes only ≈8 min.^[^ ^8^ ^]^ Consequently, although the digital SERS‐based LFIAs may exhibit lower multiplexing capabilities, they are simpler to perform, offer significantly higher sensitivity, and have a reduced overall sample‐to‐answer time.

## 3 Conclusions

In summary, we implemented a digital counting protocol to increase the analytical sensitivity of a SERS‐based LFIA for the ultrasensitive detection of SARS‐CoV‐2 in spiked nasal swabs. In comparison with the colorimetric and the average SERS intensity‐based LFIA, the LOD achieved for the detection of the nucleocapsid N protein was improved by 7 and 2 orders of magnitude, respectively. Importantly, our digital approach showed an analytical sensitivity of 0.03 TCID_50_ mL^−1^, which is far greater than that reported by other types of immunoassays. Whereas colorimetric tests can only detect the viral N protein after symptom appearance, the high sensitivity shown by the proposed method could enable an early detection of the virus, lowering the probability of further spreading an infection and contributing to improving the surveillance of the disease. Lastly, the robust and sensitive detection of SARS‐CoV2 N protein at ultralow concentration by the digital SERS‐based LFIA reported here demonstrates that this technology could be successfully applied for the detection of disease biomarkers, proteins involved in neurological disorders, and microbial pathogens at low concentrations to improve early diagnosis and treatment of disease.

## 4 Experimental Section

4.1

4.1.1

##### Materials

Anti‐SARS‐CoV2 Nucleocapsid antibodies (NUN‐CH15, AM223) and (NUN‐CH14, AS95) were purchased from Acrobiosystems. Inactivated SARS‐CoV2 virus NR‐52 287 was purchased from BEI Resources. Protein G was purchased from GenScript. BSA (≥98%), Polyvinylpyrrolidone (PVP k25, 24000), sucrose (99.5%), Triton X‐100, Phosphate buffered Saline (PBS 10x), Iron (II) sulfate heptahydrate (≥99%), silver nitrate (≥99%), sodium citrate tribasic dihydrate (≥98%), ethylenediaminetetraacetic acid tetrasodium salt hydrate (EDTA, 99%), and rhodamine B isothiocyanate (RBIT) were purchased from Sigma‐Aldrich. Hydrogen tetrachloroaurate (III) trihydrate (99.99%) was supplied by Alfa Aesar. Sulfuric acid (95–97%) was supplied by Scharlau. Citric acid monohydrate (99.5%) and sodium phosphate dibasic (≥99%) from Fluka. Nitrocellulose membranes (UniSart CN95) were purchased from Sartorius. Absorbent pads (CF6) and backing cards (10547158) were purchased from Cytiva. All chemicals were used as received, and ultrapure water (type I) was used in all the preparations. Commercially available SARS‐CoV2 were supplied from Anbio Biotech, Boson Biotech, and Sejoy.

The samples used in this study were collected from healthy subjects in full compliance with the clinical and ethical practices of the Spanish Government and the Declaration of Helsinki. The study protocol was approved by the Galician Ethics Committee (2021/353). All participants received comprehensive written and oral information prior to inclusion in the study and provided written informed consent before its commencement. Participant anonymity was maintained throughout the study.

##### Instrumentation

IsoFlow reagent dispensing system (Imagene Technology, USA) was used to dispense the control and test lines. An automatic guillotine (Samkoon, China) was used to cut the strips.

Surface‐enhanced resonance Raman scattering (SERRS) measurements were conducted with a Renishaw InVia Reflex confocal system. The spectrograph used a high‐resolution grating (1800 grooves per mm) with additional band‐pass filter optics, a confocal microscope, and a 2D‐CCD camera. SERRS images were obtained using a point‐mapping method with a 10x objective (N.A. 0.25), which provided a spatial resolution of about 5.3 μm^2^. The spectral images were obtained by measuring the SERS spectrum of each pixel of the image, one at a time, but in this case with an objective 50x (N.A. 0.40) and spatial resolution of 2.6 μm^2^. Laser excitation was carried out at 532 nm with 12.90 mW of power and 0.5 and 1 s acquisition time. The SERS images of each well were decoded using the signal to baseline area of the highest Raman peak from the reporter molecule (RBITC, 1620–1660 cm^−1^) using WiRE software V 4.1 (Renishaw, UK).

Optical characterization of the colloids was carried out using a Cary 300 UV‐Vis spectrophotometer (Varian, Salt Lake City, UT, USA). Transmission Electron Microscopy (TEM) images were acquired with a JEOL JEM 1010 TEM operating at an acceleration voltage of 100 kV.

##### Methods: Synthesis of Citrate‐Stabilized Au@Ag NPs

Au@Ag core‐shell NPs were synthesized following a previously reported method.^[^ ^54^ ^]^ Briefly, the synthesis can be divided into two steps: the synthesis of the gold seeds and the growth step forming the Au@Ag core‐shell NPs.

*Synthesis of 14.0 nm Au seeds:* 150 mL of 2.2 mM citrate buffer (75/25 sodium citrate/citric acid) was heated in a three‐neck round bottom flask until boiling point. After 15 min of boiling, EDTA was added to reach a molar concentration of 0.01 mM in the final volume. Subsequently, 1 mL of 25 mM HAuCl_4_ was added. It was allowed to react for 10 min until a pale red color was achieved and then the colloid was cooled until room temperature.

*Synthesis of Au@Ag core‐shell NPs:* First, 10 mL of 14.0 nm Au seeds (0.15 mM in Au^0^) were mixed with sodium citrate final concentration 4.4 mM, 40 μL of 1 M H_2_SO_4_, and ultrapure water until reaching 15 mL (final volume). The final pH was 4.0. The Au seed growth was performed in multiple steps. In the first overgrowth, to the Au seeds solution, 15 mL of 1 mM AgNO_3_ and 15 mL of reducing solution containing 4 mM FeSO_4_ and 4 mM sodium citrate were simultaneously added using a double syringe pump at 90 mL h^−1^. After finishing the addition of the reactants (10 min), the Ag growth is complete. Finally, sodium citrate (final concentration 2.2 mM) was added to improve the colloidal stability. In a second overgrowth step, the protocol was the same as for the first overgrowth, but using as seeds 15 mL of Au@Ag colloids obtained in the previous overgrowth step. The final nanoparticle size was ≈54 nm. The 45.3 mL of colloid were centrifuged (1160 g × 30 min). The pellet was resuspended in 4.5 mL of 1 mM sodium citrate. The stock solution remains stable for three months if stored at 4 °C and protected from light to prevent oxidation.

##### Methods: Fabrication of SERS‐encoded NPs

To codify the Au@Ag NPs, 100 μL of the concentrated colloid (O.D ≈ 8 (1 cm cuvette)) was diluted in 625 μL of ultrapure water. After the dilution, the codification with the Raman reported was carried out by adding 200 μL of a solution of 1.0 × 10^−6^ M RBITC in ethanol mixed with vortex and kept undisturbed for 30 min. After 30 min, the codified nanoparticles were centrifuged at 1000 g × 30 min. The pellets were resuspended in 750 μL of phosphate buffer (PB) buffer 10 mM pH = 10.5.

##### Methods: Conjugation of SERS‐Tags with Anti‐N Protein Antibody

For the antibody conjugation, 3 μL (1 mg mL^−1^ in PBS 1x) of Ab‐223 antibody was added to 750 μL of previously encoded SERRS tag in PB buffer 10 mM and pH = 10.5. The colloids were mixed with a vortex and kept undisturbed at room temperature for 1 h. To block the remaining free surface of the NPs, 100 μL of BSA (10 mg mL^−1^ in PB) was added and incubated for 30 min. After incubation steps, two centrifugations at 1000 g × 30 min were done. The first centrifugation pellet was redispersed in 750 μL of PB buffer, and the second one in 50 μL of a BSA—Sucrose (1–10% w/w, respectively, in PB buffer 10 mM pH 10.5). The conjugated SERS tags should be used within 48 h since longer storing times imply a decrease in their SERS performance.

##### Methods: N Protein Production and Purification

An expression vector based on pET26 (Merck Millipore) and containing the gene encoding the N protein of SARS‐CoV2 was a kind gift of Hugh Reyburn (CNB‐CSIC, Madrid).^[^ ^76^ ^]^ The above‐mentioned plasmid was transformed into the *E. coli* BL21(DE3) (Thermo Fisher Scientific) and grown overnight at 37 °C in 40 mL of LB media containing kanamycin (50 μg mL^−1^) and shaking at 150 rpm. The bacterial culture was inoculated in 4L of LB media with the same antibiotic concentration and grown at 37 °C until the bacterial culture reached an optical density of 0.4–0.5 at 600 nm. The culture was then cooled on ice to 22 °C, IPTG was added to a final concentration of 1 mM, and the culture was incubated overnight at 22 °C and with shaking (150 rpm). The next day, bacteria were harvested by centrifugation and stored at −20 °C until use.

For protein purification, pellets from 4 L of culture were resuspended in 80 mL TN400G buffer (50 mM Tris‐HCl pH 8, 400 mM NaCl, 5% v/v glycerol) containing DNAse and RNAse added to final concentrations of 50 μg mL^−1^ and a mixture of protease inhibitors [Sigma‐Aldrich]) and then cooled on ice for 15 min. Bacteria were lysed by sonication, using four cycles of 30 s with 30 s rest on ice between pulses and 40% sonic power (Branson Model 102C sonifier). Bacterial debris was removed by centrifugation at 11 000 × g at 4 °C for 1 h and the supernatant was filtered through a 45 μm syringe filter. The 6‐histidine tagged protein was purified from the lysate by steps of affinity and size exclusion chromatography. The bacterial supernatant was incubated with 2 mL of Ni‐NTA resin (Jena Bioscience) for 1 h on ice and passed through the column at room temperature, followed by washing with 10 column volumes of washing buffer B (25 mM imidazole in TN400G buffer) and 5 column volumes of washing buffer C (50 mM imidazole in TN400G buffer). The recombinant protein was then eluted with buffer D (250 mM imidazole in TN400G buffer). Afterward, the purified protein was dialyzed o/n against 50 mM Tris pH 8, 0.2 M NaCl, and 5% v/v glycerol (TN200G buffer) at 4 °C to reduce the salt concentration. Size exclusion chromatography was carried out using a Hiload Superdex 200 16/60 column (Cytiva) previously equilibrated with TN200G buffer 2 mL min^−1^. Fractions were collected and dialyzed against PBS. Protein purity and concentration were estimated by SDS‐PAGE and UV absorbance (A280/A260 ratio).

##### Methods: Fabrication of LFIA Strips

To fabricate the strip, the nitrocellulose membrane was attached to a plastic backing card. The control line of the strips was prepared by dispensing 1 mg mL^−1^ of protein G. The test line was prepared by dispensing 1.0 mg mL^−1^ of Ab‐95. The established order of the lines was control line (line above) and test line (line below). All the lines were dispensed with the IsoFlow dispenser onto a nitrocellulose membrane at a dispensing ratio of 0.100 μL mm^−1^. The strips were dried at 37 °C for 30 min. The absorbent pad was attached to the end of the membrane on the backing card with an overlap between them of around 2.5 mm. The complete strip was cut into individual 5 mm strips. The strips were used up to three months after they were manufactured without any effect on their performance efficiency.

##### Methods: N Protein and Virus Spike Calibration Curves Procedure

Different concentrations of N protein (1 × 10^4^–1 × 10^−5^ pg mL^−1^) were spiked into nasal swabs obtained from healthy volunteers. The same was done for the inactivated virus, and different viral loads (1 × 10^5^–1 × 10^−3^ TCID_50_ mL^−1^) were spiked onto the nasal swabs. Subsequently, the samples were mixed, in a 96‐well assay plate, with 80 μL of running buffer (3% BSA + 1% PVP + 0.5% Triton X‐100 in PBS), 10 μL of the as‐prepared sample and 10 μL of SERS‐tags (OD ≈ 10 after concentration). After 20 min, 30 μL of the running buffer was added to the strip to clean it, and after 10 min, the strips were dried with compressed air and the absorbents were removed from the strips to stop the flow to avoid non‐specificities that could happen due to backflow of the nanoparticles from the absorbent.

##### Methods: Analog and Digital SERS Analysis

SERS mapping was measured over a 3100 × 1300 μm^2^ area (403 points, 100 μm step size) measured with a 532 nm laser line, 50× objective, 12.90 mW laser power, and an acquisition time of 1 s. For the analog SERS protocol, the peak intensity at 1645 cm^−1^ was measured for both N protein and virus calibrations. In contrast, for the digital SERS protocol, the signal‐to‐baseline SERS area mappings between 1620 and 1660 cm^−1^ were converted to binary format by applying a specific threshold. This threshold was determined based on the average area plus 3 times the standard deviation above the mean measured in the control test without antigen (blank). Pixels with signal‐to‐baseline SERS areas between 1620 and 1660 cm^−1^ above the threshold were assigned a value of 1 (black squares), while those equal to or below the threshold were set to 0 (white squares). Further details on data treatment and analysis can be found in Figure S4.

## Conflict of Interest

The authors declare no conflict of interest.

## Author Contributions

**Lara González‐Cabaleiro**: Formal analysis (lead); Investigation (lead); Validation (lead). **Carlos Fernández‐Lodeiro**: Investigation (equal). **Lorena Vázquez‐Iglesias**: Investigation (equal); Validation (equal); Writing—original draft (lead). **Gustavo Bodelón**: Conceptualization (equal); Writing—original draft (lead); Writing—review and editing (supporting). **Pablo Soriano‐Maldonado**: Investigation (supporting). **Mark J. van Raaij**: Investigation (supporting). **Isabel Pastoriza‐Santos**: Conceptualization (lead); Funding acquisition (lead); Resources (lead); Supervision (lead); Writing—original draft (lead); Writing—review and editing (lead). **Jorge Pérez‐Juste**: Conceptualization (lead); Formal analysis (equal); Funding acquisition (lead); Project administration (lead); Resources (lead); Writing—original draft (lead); Writing—review and editing (lead).

## Supporting information

Supplementary Material

## Data Availability

The data that support the findings of this study are openly available in Zenodo at https://doi.org/10.5281, reference number 11275801.
