# Supplementary material for: Pushing the Limits of Lateral Flow Immunoassay by Digital SERS for the Ultralow Detection of SARS‐CoV‐2 Virus
Source: Small Sci. 2024 Aug 10;4(11):2400259. doi: 10.1002/smsc.202400259 (PMC11935054; doi:10.1002/smsc.202400259)
Supplement: Supplementary file 1 — Supplementary Material [file SMSC-4-2400259-s001.pdf]

Supporting Information

for

Pushing the limits of lateral flow immunoassay by Digital SERS for the ultralow detection of SARS-CoV-2 virus

Pushing the limits of SARS-CoV-2 detection by digital SERS lateral flow immunoassay

Lara González-Cabaleiro,[a,b] Carlos Fernández-Lodeiro,[a,b] Lorena Vázquez-Iglesias,[a] Pablo Soriano-Maldonado,[c,d] Mark J. van Raaij,[c] Gustavo Bodelón,[a,e,*] Jorge Pérez-Juste,[a,b,*] and Isabel Pastoriza-Santos[a,b,*]

[a] CINBIO, Universidade de Vigo, Campus Universitario As Lagoas, Marcosende, 36310 Vigo, Spain

[b] Departamento de Química Física, Universidade de Vigo, Campus Universitario As Lagoas, Marcosende, 36310 Vigo, Spain.

[c] Centro Nacional de Biotecnología (CNB-CSIC), calle Darwin 3, 28049 Madrid, Spain.

[d] Faculty of Experimental Sciences, Universidad Francisco de Vitoria (UFV), 28223. Pozuelo de Alarcón Madrid, Spain.

[e] Departamento de Biología Funcional y Ciencias de la Salud, Universidade de Vigo, Campus Universitario As Lagoas, Marcosende, 36310 Vigo, Spain.

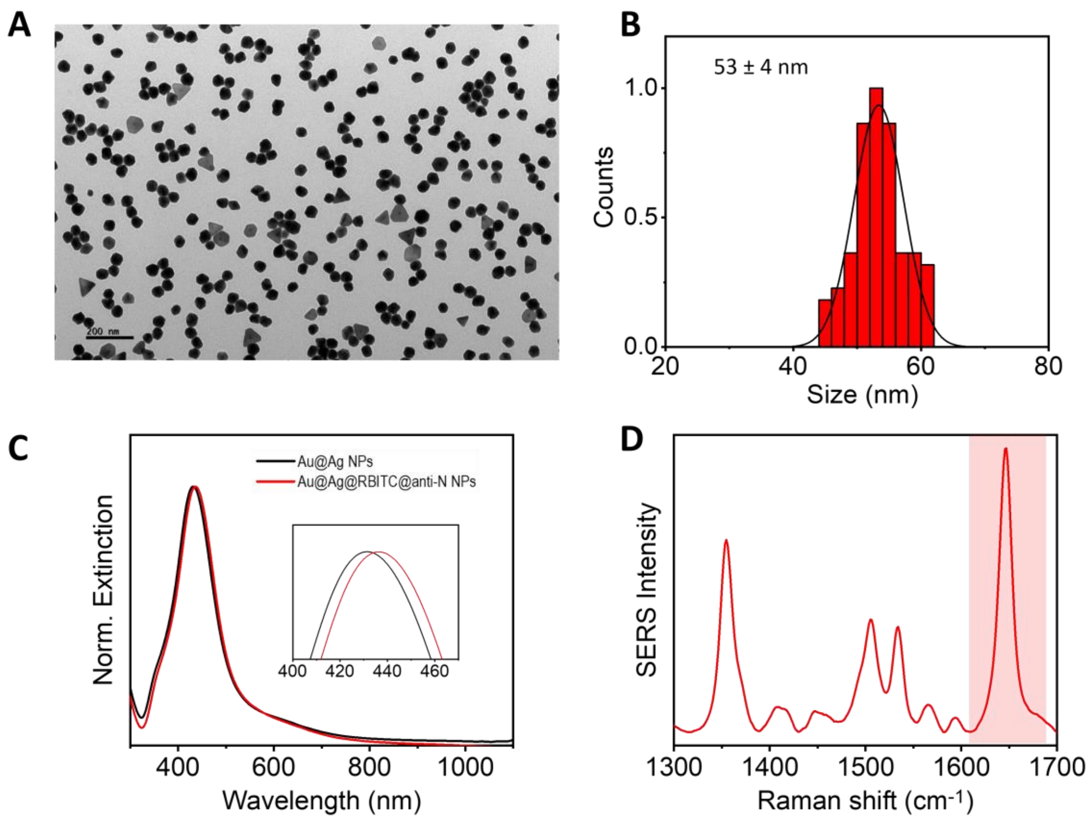

Figure S1. SERS tags characterization. (A) Representative TEM image of spherical Au@Ag core-shell nanoparticles (Au@Ag NPs). (B) Size distribution histogram of Au@Ag NPs. (C) Normalized extinction spectra of Au@Ag NPs before (black) and after functionalization with RBITC and anti-N-protein (red line). The inset clearly shows the red shift in the plasmon band peak after the codification of Au@Ag NPs. (D) SERS spectrum of the SERS tags encoded with RBITC. The red shadowed region indicates the 1645 cm^{-1} Raman peak of RBITC-encoded SERS tag.

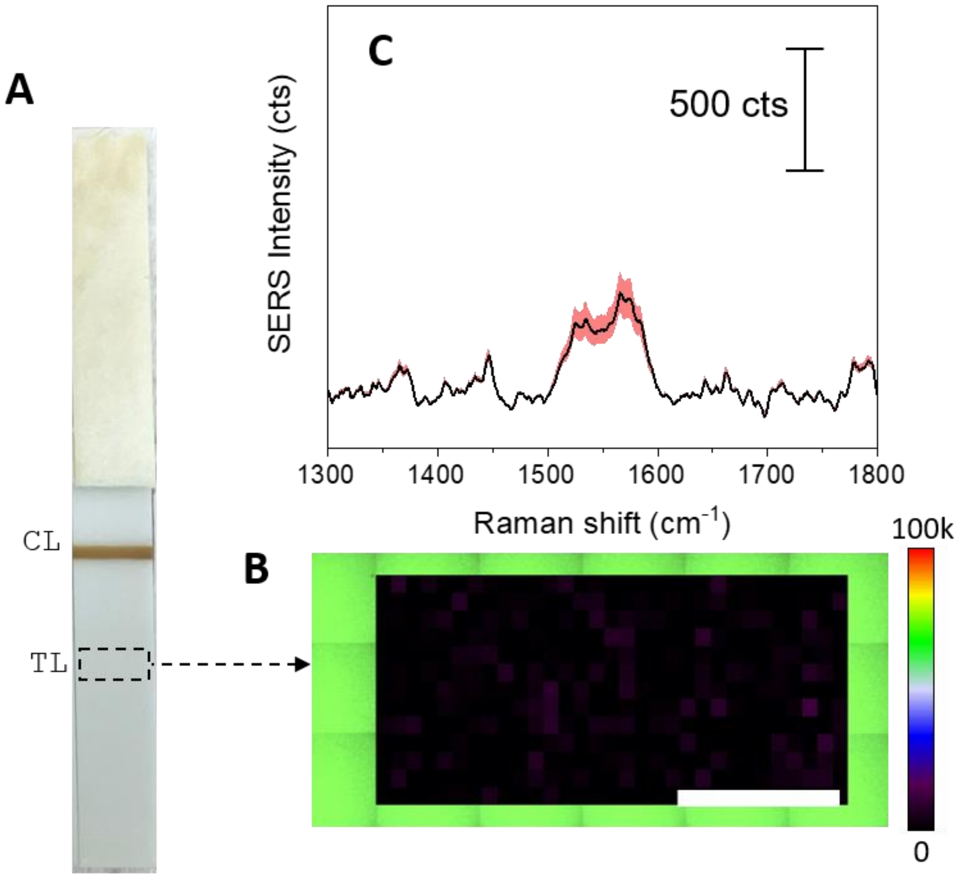

Figure S2. Optimization of the assay in the absence of N protein. (A) LFIA strip photograph in the absence of protein N. The dashed rectangle in the test line shows the region of the strip measured by SERS. (B) Signal to baseline SERS mappings were acquired between 1620-1660 cm^{-1} over an extended area of 3100 × 1300 μm^2 with a 532 nm laser line, 50× objective, 12.90 mW laser power, acquisition time 1 s, 100 µm step size and 403 points. The scale bar in white represents 100 µm. (C) Figure shows the mean SERS spectrum as well as the standard deviation of the SERS mapping showing the absence of the characteristic bands of RBITC and demonstrating the absence of non-specificities in the assay.

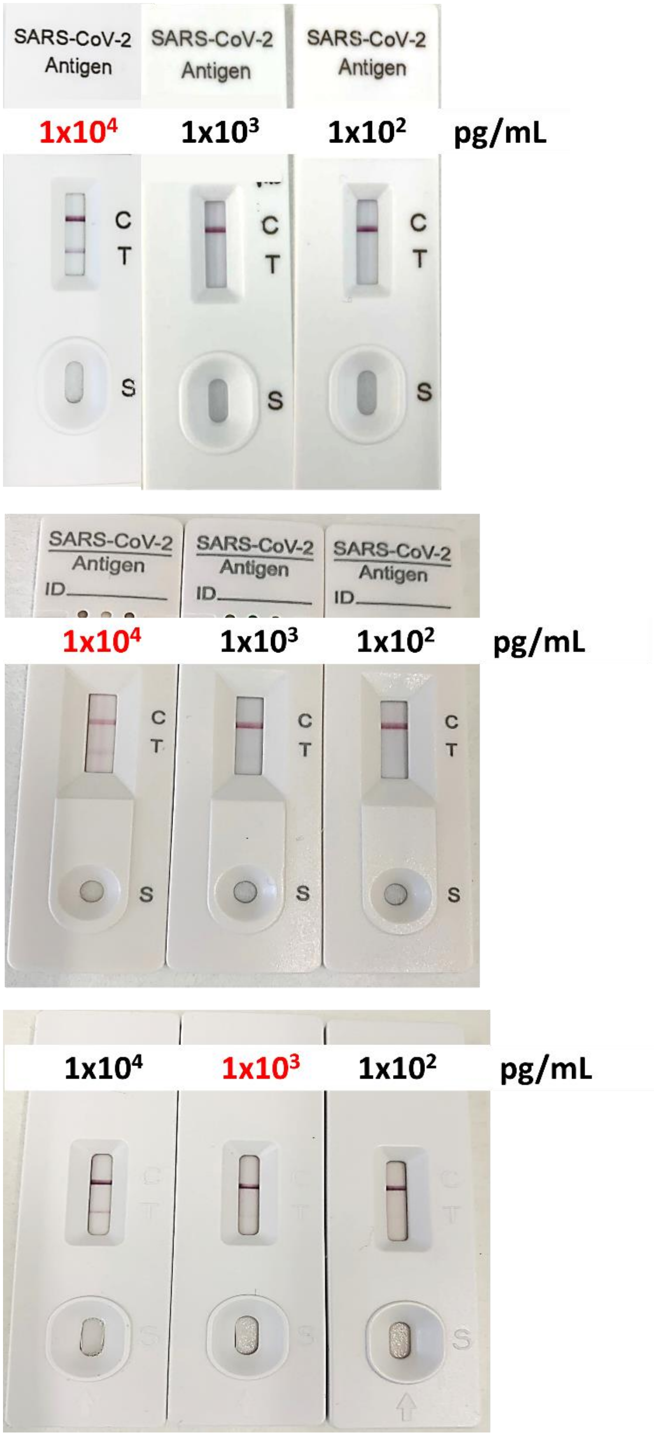

Figure S3. Photographs of commercial colorimetric antigen test (Anbio Biotech, Boson Biotech and Sejoy) with different concentrations of the recombinant N protein used for the digital calibration curve. The LOD obtained was either 1000 pg/mL or 10000 pg/mL depending on the test.

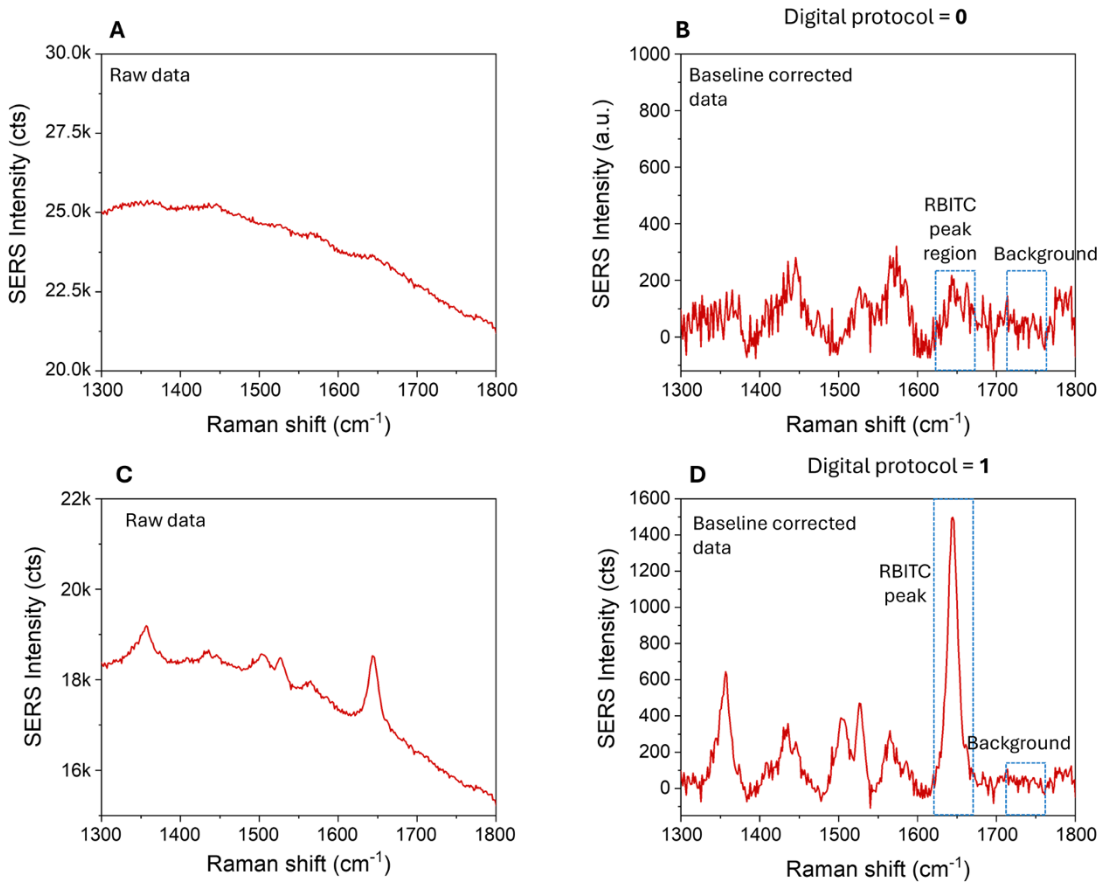

Figure S4. Digital protocol. Figures S4A and **S4B** show a representative raw data spectrum for a negative count and a positive count, respectively. The first step in the data treatment is a baseline correction of the spectra using the Savitsky-Golay method. **Figures S4C** and **S4D** show the spectra of the curves for S3A and S3B after baseline correction, respectively. Afterwards, the signal to base intensity between 1620 cm^{-1} and 1660 cm^{-1} is calculated for both spectra. If the peak area signal to base intensity is higher than three times the background the value is considered as 1 (positive event), if the area is equal or smaller than three times the background is considered as 0 (negative event).

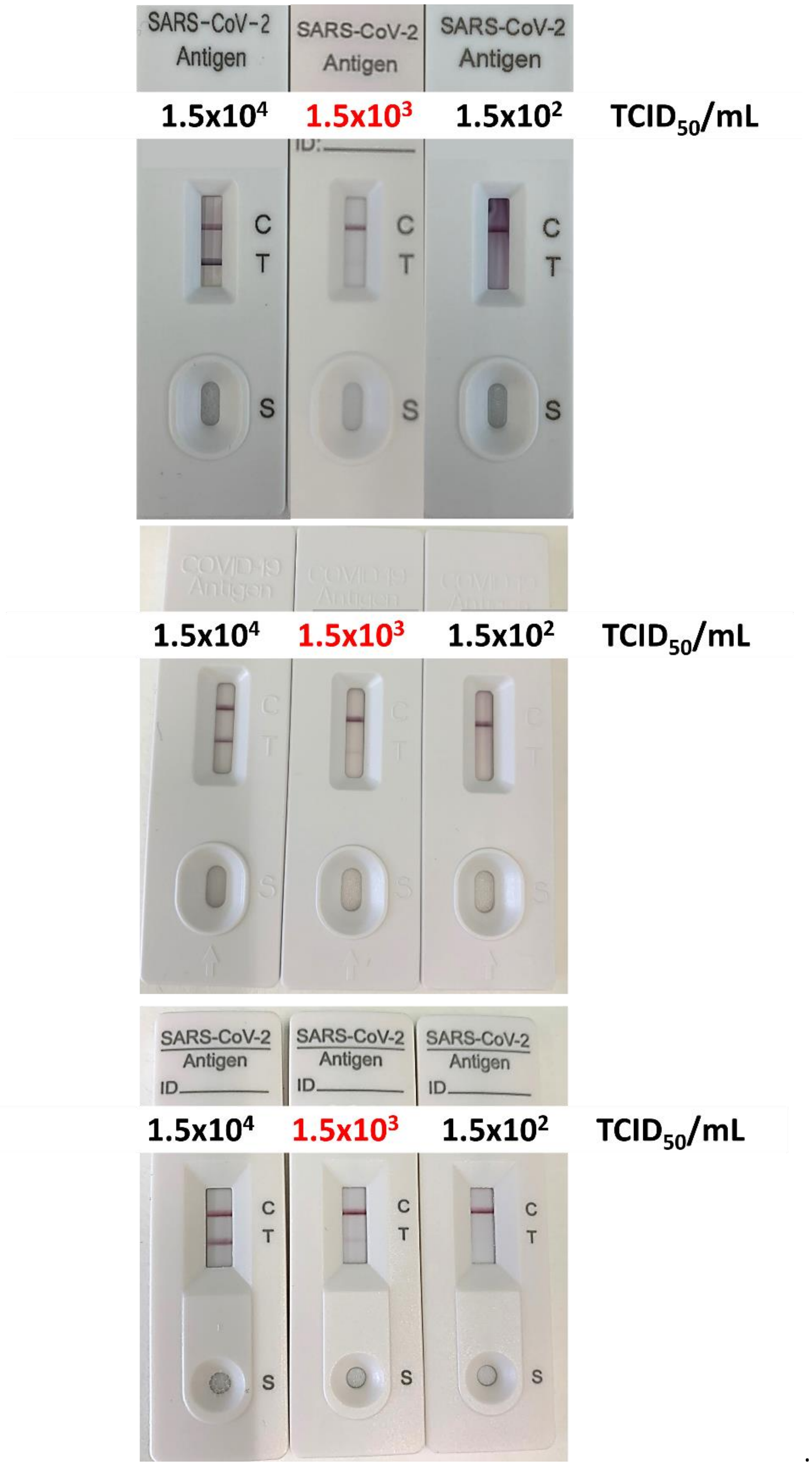

Figure S5. Photographs of commercial colorimetric antigen test (Anbio Biotech, Boson Biotech and Sejoy) with different viral load of the inactivated virus used for the digital calibration curve. The LOD obtained was 10^3 $TCID_{50}$/mL.

Table S1. Comparison of SERS-based LFIAs and SERS-based digital immunoassays for the detection of SARS-CoV-2 N and S proteins.

Method	Nanostructures	LOD	Protein	Sample	Ref.
SERS-LFIA	$Au@^{4\text{-}MBA}@Ag$ NPs	0.03 ng/mL	N	Spiked in saliva	[1]
SERS-LFIA	Ag/BP^{RhB} nanosheets	0.5 pg/mL	N	Diluted in buffer	[2]
SERS-LFIA	$Au@^{4\text{-}MBA}$ NPs	0.1 ng/mL	S	Diluted in buffer	[3]
Digital SERS-LFIA	$Au^{4\text{-}MBA}@Ag$ NPs	12 pg/mL	N	Diluted in buffer	[4]
Digital SERS immunoassay	$Au^{4\text{-}MBA}@Ag$ NPs	6.3 ng/mL	S	Spiked in diluted saliva	[5]
Digital SERS immunoassay	$Au@^{4\text{-}NBT}$ NPs	19 fg/mL	S	Diluted in buffer	[6]
Digital SERS-LFIA	$Au^{RBITC}@Ag$ NPs	0.9 fg/mL	N	Spiked in nasal swab	This work

Table S2. Reported methods and LODs for SARS-CoV-2 immunoassays.

Method	Detection	$TCID_{50}$/mL	TCID Sample	Protein:LOD	Sample	Reference
LFIA	Colorimetric	10^2-10^5	Spiked PBS	n.d.	n.d.	[7]
			Spiked PBS	n.d.	n.d.	[8]
		109-911	Spiked DMEM	N: 0.013-0.109 ng/mL	Lysis buffer	[9]
		4.44 x10^2	Spiked nasal fluid	n.d.	n.d.	Swab-N-Go[1] [10]
		140.6	Nasal swab	n.d.	n.d.	BinaxNOW[2] [11]
		91.7	Nasal swab	n.d.	n.d.	Sofia 2 Flu + SARS[3] [12]
		4.9	Viral culture lysate	N: 0.24 pg/mL	Assay buffer	[13]
Fluorescent microsphere immunochromatography	Fluorescence	10^3	Oropharyngeal swabs	N:100 ng/mL	n.d.	[14]
ELISA	Chemiluminescence	140	n.d.	S: 147 pg/mL	PBS	[15]
		15	Viral culture lysate	N: 2.5 ng/mL	Spiked in nasopharyngeal sample	[16]
		n.d.	n.d.	N: 2 pg/mL	Human serum	[17]
ECLIA	Electrochemiluminescence	22.5	Spiked in buffer	n.d.	n.d.	Elecsys[4]
S-PLEX assay	Electroluminescence	0.36	n.d.	N:160 fg/mL	Nasopharyngeal sample	[18]
SERS-based microdroplet	SERS	0.32	n.d.	n.d.	n.d.	[19]
Digital Simoa	Fluorescence	0.29	Spiked nasal swab	N: 99 fg/mL	Nasopharyngeal swab	Simoa[5,6]
Digital ULISA	Fluorescence	0.08	Viral culture lysate	N: 330 fg/mL	n.d.	[20]
Digital SERS LFIA	SERS	0.03	Spiked nasal swab	N: 0.9 fg/mL	Spiked nasal swab	This work

[1] fda.gov/media/170419/download
[2] fda.gov/media/141570/download
[3] fda.gov/media/142704/download
[4] diagnostics.roche.com/es/es/products/params/elecsys-sars-cov-2-antigen-test.html
[5] quanterix.com/wp-content/uploads/2021/05/IFU-0002v11_5.24.21
[6] quanterix.com/wp-content/uploads/2020/12/SARS-CoV-2-N-Protein-Advantage-Data-Sheet-for-HD-X

References

[1] S. Lai, Y. Liu, S. Fang, Q. Wu, M. Fan, D. Lin, J. Lin, S. Feng, *J. Biophotonics* **2023**, *16*, e202300004.

[2] C. Lin, Z. Liu, F. Fang, S. Zhao, Y. Li, M. Xu, Y. Peng, H. Chen, F. Yuan, W. Zhang, X. Zhang, Z. Teng, R. Xiao, Y. Yang, *ACS Sens.* **2023**, *8*, 3733.

[3] K. V. Serebrennikova, N. A. Byzova, A. V. Zherdev, N. G. Khlebtsov, B. N. Khlebtsov, S. F. Biketov, B. B. Dzantiev, *Biosensors (Basel)* **2021**, *11*, 510.

[4] W. Wang, S. Srivastava, A. Garg, C. Xiao, S. Hawks, J. Pan, N. Duggal, G. Isaacman-VanWertz, W. Zhou, L. C. Marr, P. J. Vikesland, *Environ. Sci. Technol.* **2023**, *58*, 4926.

[5] A. Tuckmantel Bido, A. G. Brolo, *ACS Appl. Nano Mater.* **2023**, *6*, 15426.

[6] J. E. Shim, Y. J. Kim, J. H. Choe, T. G. Lee, E. A. You, *ACS Appl. Mater. Interfaces* **2022**, *14*, 38459.

[7] Y. Morinaga, H. Yamada, Y. Yoshida, H. Kawasuji, Y. Yamamoto, *J. Infect. Chemother.* **2023**, *29*, 131.

[8] S. Stanley, D. J. Hamel, I. D. Wolf, S. Riedel, S. Dutta, E. Contreras, C. J. Callahan, A. Cheng, R. Arnaout, J. E. Kirby, P. J. Kanki, *J. Clin. Microbiol.* **2022**, *60*, e00140.

[9] T. Peng, L. Dong, X. Feng, Y. Yang, X. Wang, C. Niu, Z. Liang, W. Qu, Q. Zou, X. Dai, M. Li, X. Fang, *Talanta* **2023**, *258*, 124462.

[10] I. Inc, *Rapid Diagnostic Test for Detection of SARS-CoV-2 Antigen For Emergency Use Authorization (EUA) Only For Use with Anterior Nasal Swab Specimens For in Vitro Diagnostic Use Rapid Diagnostic Test for the Detection of SARS-CoV-2 Antigen HEALTHCARE PROVIDER INSTRUCTIONS FOR USE*, **2023**.

[11] *PROCEDURE CARD For Use Under an Emergency Use Authorization (EUA) Only. Part 1-Sample Test Procedure Patient Samples Require 6 Drops of Extraction Reagent*, **2023**.

[12] *Sofia 2 Flu + SARS Antigen FIA - Instructions For Use*, **n.d.**

[13] D. Hong, E. J. Jo, C. Jung, M. G. Kim, *ACS Appl. Mater. Interfaces* **2022**, *14*, 45189.

[14] C. Zhang, L. Zhou, K. Du, Y. Zhang, J. Wang, L. Chen, Y. Lyu, J. Li, H. Liu, J. Huo, F. Li, J. Wang, P. Sang, S. Lin, Y. Xiao, K. Zhang, K. He, *Front. Cell Infect. Microbiol.* **2020**, *10*, 553837.

[15] G. C. Girt, A. Lakshminarayanan, J. Huo, J. Dormon, C. Norman, B. Afrough, A. Harding, W. James, R. J. Owens, J. H. Naismith, *R. Soc. Open Sci.* **2021**, *8*, 211016.

[16] S. K. P. Lau, P. C. Y. Woo, B. H. L. Wong, H. W. Tsoi, G. K. S. Woo, R. W. S. Poon, K. H. Chan, W. I. Wei, J. S. Malik Peiris, K. Y. Yuen, *J. Clin. Microbiol.* **2004**, *42*, 2884.

[17] R. F. Thudium, M. P. Stoico, E. Høgdall, J. Høgh, H. B. Krarup, M. A. H. Larsen, P. H. Madsen, S. D. Nielsen, S. R. Ostrowski, A. Palombini, D. B. Rasmussen, N. T. Foged, *J. Clin. Microbiol.* **2021**, *59*, e01001.

[18] N. R. Pollock, T. J. Savage, H. Wardell, R. A. Lee, A. Mathew, M. Stengelin, G. B. Sigal, *Am. Soc. Microbiol.* **2021**, *59*, 03077.

[19] S. Park, C. Su Jeon, N. Choi, J. Il Moon, K. Min Lee, S. Hyun Pyun, T. Kang, J. Choo, *Chem. Eng. J.* **2022**, *446*, 137085.

[20] J. C. Brandmeier, N. Jurga, T. Grzyb, A. Hlaváček, R. Obořilová, P. Skládal, Z. Farka, H. H. Gorris, *Anal. Chem.* **2023**, *95*, 4753.
